# Inactive rhomboid proteins RHBDF1 and RHBDF2 (iRhoms): a decade of research in murine models

**DOI:** 10.1007/s00335-021-09910-3

**Published:** 2021-09-03

**Authors:** Lisa M. Burzenski, Benjamin E. Low, Vivek Kohar, Leonard D. Shultz, Michael V. Wiles, Vishnu Hosur

**Affiliations:** The Jackson Laboratory, Bar Harbor, ME USA

## Abstract

Rhomboid proteases, first discovered in *Drosophila*, are intramembrane serine proteases. Members of the rhomboid protein family that are catalytically deficient are known as inactive rhomboids (iRhoms). iRhoms have been implicated in wound healing, cancer, and neurological disorders such as Alzheimer’s and Parkinson’s diseases, inflammation, and skin diseases. The past decade of mouse research has shed new light on two key protein domains of iRhoms—the cytosolic N-terminal domain and the transmembrane dormant peptidase domain—suggesting new ways to target multiple intracellular signaling pathways. This review focuses on recent advances in uncovering the unique functions of iRhom protein domains in normal growth and development, growth factor signaling, and inflammation, with a perspective on future therapeutic opportunities.

## The rhomboid proteins: rhomboid proteases and rhomboid pseudoproteases

**Fig. 1:**
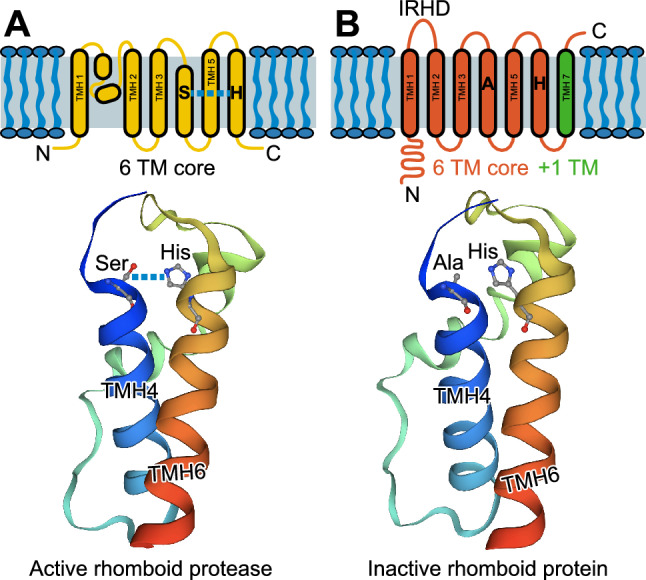
Schematic topological models of the Rhomboid proteins. The rhomboid proteins: both active and inactive rhomboid proteins contain a cytosolic N-terminal domain (N), a six or seven transmembrane domain (TMD), an extracellular loop or iRhom homology domain (IRHD) (in the case of iRhoms) in between transmembrane helices (TMH) 1 and 2, and a C-terminal domain (C). **A** In the case of active rhomboids, the catalytic dyad is formed by the highly conserved serine and histidine residues in transmembrane helices 4 and 6, respectively. **B** iRhoms lack the serine residue in transmembrane helix 4 and hence lack serine protease activity. The *E. coli* rhomboid protease GlpG crystal structure shows the catalytic dyad residues serine and histidine (left) in transmembrane helices 4 and 6. Catalytic serine has been replaced with alanine in iRhoms in transmembrane helix 4 (right)

Rhomboid proteins are a highly conserved superfamily of polytopic membrane proteins (Urban and Dickey 2011). Rhomboid proteins can be broadly classified into active (Lastun et al. 2016) and inactive enzymes (Freeman 2014), also called pseudoproteases or iRhoms. Active rhomboid proteins, first discovered in *Drosophila* as key regulators of epidermal growth factor receptor (EGFR) signaling, are intramembrane serine proteases that hydrolyze peptide bonds within the lipid bilayer (Lemberg et al. 2005). Catalysis is achieved by a histidine-serine dyad, which is submerged 10 Å below the cell membrane surface (Fig. 1A). Conversely, iRhoms lack a catalytic serine residue and hence do not retain any enzymatic protease activity (Fig. 1B). Nevertheless, the iRhoms RHBDF1 and RHBDF2 have been implicated in neurological disorders including Alzheimer’s and Parkinson’s diseases, as well as in cancer, inflammation, and skin diseases (Jager et al. 2014; Raj et al. 2018; Hosur et al. 2014; Hosur et al. 2018; Yan et al. 2008; Zhou et al. 2014; Zou et al. 2009; Blaydon et al. 2012; Young 2019). Nearly a decade of research in mice has emphasized an essential role for these two proteins in normal functioning of the brain, heart, skin, eye, bone, adipose tissue, and the immune system. In line with the role of active rhomboids in EGFR signaling, findings from mouse models demonstrate that iRhoms are also essential regulators of EGFR signaling. While loss-of-function (LOF) mutations in *Rhbdf1*, *Rhbdf2,* or both significantly suppress stimulated secretion of EGFR ligands, gain-of-function (GOF) mutations in either *Rhbdf1* or *Rhbdf2* stimulate enhanced EGFR ligand secretion. iRhoms consist of a long cytosolic N-terminal domain, a conserved cysteine-rich iRhom homology domain (IRHD), a six transmembrane (TM helices 1–6) core (Fig. 1B), and an additional TM segment (TM helix 7). The six TM core harbors the dormant peptidase domain (TM helices 2–6), which has an alanine residue (instead of serine) in the enzyme core. In this review, we describe how insights from mouse models carrying either spontaneous mutations or CRISPR/Cas9-induced gene modifications in *Rhbdf1* and *Rhbdf2* have been crucial in identifying their physiological targets, in defining the unique roles of iRhom protein domains in development and disease, and in nominating possible novel therapeutic opportunities targeting iRhoms.

## Overlapping and discrete functions of RHBDF1 and RHBDF2 in regulating EGFR signaling

### Evidence for shared targets and function

**Fig. 2:**
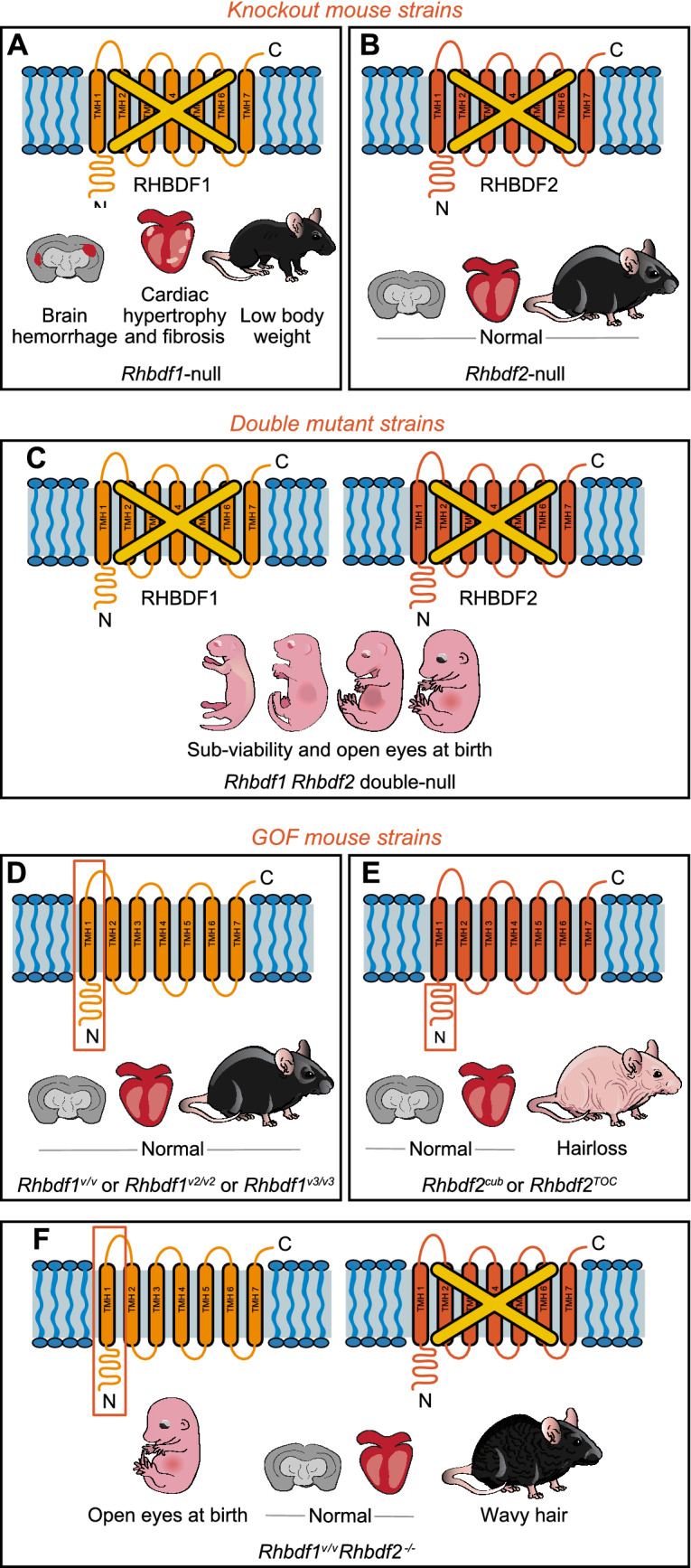
Loss-of-function (LOF) and gain-of-function (GOF) mouse models of iRhoms. **A** *Rhbdf1-*null mice exhibit brain hemorrhage, cardiac fibrosis, and lower body weight than control littermates. **B** *Rhbdf2-null* mice are healthy and fertile and do not show brain, heart, or growth defects. **C** A combined loss of *Rhbdf1* and *Rhbdf2* results in sub-viability and eyelids open at birth (EOB) phenotype. **D** GOF mutations in the *Rhbdf1* gene, such as viable (*v*) 1, viable 2, and viable 3, produce an N-terminal-truncated protein to rescue the overt phenotype observed in the *Rhbdf1-*null mice in panel A. **E** GOF mutations in the *Rhbdf2* gene, such as *Rhbdf2*^*cub*^, *Rhbdf2*^*uncv*^, and *Rhbdf2*^*P*159*L*^, exhibit a loss-of-hair phenotype through enhanced secretion of EGFR ligand AREG via the TMD. **F** The *Rhbdf1*^*v/v*^ allele reverses sub-viability of *Rhbdf1:Rhbdf2* double KO mice in panel C. *Rhbdf1*^*v/v*^*Rhbdf2*^*−/−*^ mice show an EOB phenotype and develop a wavy hair coat; however, no cardiac or brain abnormalities are observed in *Rhbdf1*^*v/v*^*Rhbdf2*^*−/−*^ double mutant mice. Rectangles indicate deleted regions

Homozygous *Rhbdf1* knockout mice (KO) exhibit multiorgan pathologies, including brain hemorrhage, cardiac fibrosis, and lower body weight compared with heterozygous littermates, and die within two weeks after birth (Christova et al. 2013; Hosur et al. 2020) (Fig. 2A). Conversely, *Rhbdf2-null* mice are healthy and fertile and do not present with growth retardation or brain and heart defects (Hosur et al. 2014; Adrain et al. 2012; McIlwain et al. 2012; Siggs et al. 2012) (Fig. 2B). Nevertheless, a combined absence of RHBDF1 and RHBDF2 results in a more severe phenotype than either *Rhbdf1* or *Rhbdf2* KO, as evidenced by sub-viability and eyelids open at birth (EOB) observed in *Rhbdf1:Rhbdf2* double KO mice (Fig. 2C) (Hosur et al. 2020). This phenotype, together with the multiorgan pathology exhibited by *Rhbdf1*-null mice, suggests that (1) RHBDF1 and RHBDF2 have overlapping functions, as the presence of RHBDF2 reverses sub-viability and the EOB phenotype in *Rhbdf1* KO mice (Hosur et al. 2020), and (2) RHBDF1 and RHBDF2 share some physiological functions and targets.

### Evidence for distinct targets and function

Studies of *Rhbdf1* and *Rhbdf2* GOF mutant mice (Fig. 2D, E)—*Rhbdf1*^*viable*^ (Hosur et al. 2020), curly bare (*Rhbdf2*^*cub*^), uncovered (*Rhbdf2*^*uncv*^), and CRISPR/Cas9-mediated genetically engineered mouse model of tylosis with esophageal cancer (*Rhbdf2*^*TOC*^)—suggest that RHBDF1 and RHBDF2 have distinct functions, and thereby distinct physiological targets. *Rhbdf1*^*viable*^ mice, which are generated using CRISPR/Cas9-mediated excision of exons 2 and 3 of the *Rhbdf1* gene, are healthy, viable, and fertile. Despite lacking the exons containing the transcription start site (ATG), the mutant *viable* transcript produces an N-terminal-truncated RHBDF1 protein (ΔN1–151) using the next in-frame ATG, which is in exon 4. While most *Rhbdf1-*null mice die by postnatal day 14, no abnormalities are observed in *Rhbdf1*^*viable*^ mice (Fig. 2D), suggesting that the *viable* mutation rescues the severe multiorgan pathologies observed in RHBDF1-deficient mice. Additionally, the *viable* allele rescues the sub-viability of *Rhbdf1:Rhbdf2* double KO mice (Fig. 2F) and induces enhanced stimulated secretion of EGFR ligands in vitro, suggesting that *viable* is a gain-of-function mutation. Consistent with the observations that mutations in the N-terminus of *Rhbdf1* result in GOF mutants, either loss of the N-terminus (loss of amino acids 1–268 [*ΔN1–268*], *Rhbdf2*^*cub*^ mutation) (Hosur et al. 2014; Siggs et al. 2014), or missense mutations (p.P159L, *Rhbdf2*^*TOC*^ mutation) (Hosur et al. 2017) in the N-terminus of *Rhbdf2*, yields GOF mutant mice. Each of these two mutations induces, through enhanced amphiregulin (AREG) secretion, accelerated wound healing and a loss-of-hair phenotype (Fig. 2E). In addition, *uncovered,* a recessive mouse mutation (*Rhbdf2*^*uncv*^) results from a spontaneous loss of 309 bp in the N-terminus of *Rhbdf2* (ΔN118–191) (Li et al. 1999). Like *Rhbdf2*^*cub*^ and *Rhbdf2*^*TOC*^*,* *Rhbdf2*^*uncv*^ mice exhibit a loss-of-hair phenotype. Loss of RHBDF2 does not affect skin architecture or hair development, indicating that *Rhbdf2*^*uncv*^ is a GOF mutation and that mutations in the N-terminus of *Rhbdf2* facilitate transmembrane domain (TMD)-mediated enhanced secretion of EGFR ligands. Interestingly, whereas *Rhbdf2 GOF* (*Rhbdf2*^*cub*^, *Rhbdf2*^*TOC*^, and *Rhbdf2*^*uncv*^) mutant mice exhibit a loss-of-hair phenotype through enhanced secretion of AREG (Christova et al. 2013; Siggs et al. 2012), *Rhbdf1* GOF (*Rhbdf1*^*viable*^ and *Rhbdf1*^*viable2*^) mutant mice have a normal hair coat (Hosur et al. 2020), suggesting that RHBDF1 and RHBDF2 have distinct physiological substrates and non-overlapping phenotypes.

## Physiological targets of iRhoms

### RHBDF1

In vitro studies in mouse embryonic fibroblasts reveal that RHBDF1 deficiency suppresses the stimulated secretion of EGFR ligands, including AREG, heparin-binding EGF (HB-EGF), and transforming growth factor-alpha (TGFα) (Hosur et al. 2020; Li et al. 2015). Additionally, short hairpin RNA (shRNA) or small interfering RNA (siRNA)-mediated silencing of *RHBDF1* in various human breast cancer cell lines and a human squamous cancer cell line significantly inhibited TGFα-mediated EGFR signaling and, further, showed anti-cancer effects by inhibiting cell proliferation and invasion and, ultimately, tumor growth in vivo (Yan et al. 2008; Zhou et al. 2014; Zou et al. 2009). These studies suggest that RHBDF1 might regulate EGFR signaling through secretion of multiple EGFR ligands, and that RHBDF2 does not compensate for the loss of RHBDF1-mediated signaling underlying the multiorgan pathologies observed in *Rhbdf1* KO mice.

Despite the clear biological importance of RHBDF1, the precise molecular mechanisms and the physiological targets of RHBDF1 underlying the multiorgan pathology and anti-cancer effects resulting from RHBDF1 deficiency remain to be investigated. It is unlikely that the pathology observed in *Rhbdf1-*null mice is mediated solely through AREG, HB-EGF, and/or TGFα, as mice lacking either AREG or TGFα are healthy and fertile (Luetteke et al. 1999; Luetteke et al. 1993; Mann et al. 1993). However, RHBDF1 likely regulates the secretion of more than one EGFR ligand. In particular, the cardiac fibrosis observed in *Rhbdf1-*null mice resembles the heart enlargement displayed by *Hbegf*-null mice (Iwamoto et al. 2003), and the eyelids open at birth (EOB) observed in *Rhbdf1:Rhbdf2* double KO mice resembles the EOB phenotype displayed by *Hbegf* and *Tgfa* double null mice (Mine et al. 2005). Together, these observations suggest that HB-EGF and TGFα could be the physiological targets of RHBDF1.

If defects in EGFR signaling alone underlie the multiorgan pathology observed in *Rhbdf1-*null mice, it is likely that RHBDF2 compensates for the loss of RHDBF1 during early development through secretion of TGFα, particularly during eyelid development. This is because, although *Rhbdf1-*null mice exhibit multiorgan pathology (Hosur et al. 2020), they do not exhibit the in utero lethality, EOB phenotype, and epidermal defects observed in *Egfr-*null mice (Miettinen et al. 1999; Sibilia and Wagner 1995). Furthermore, mice lacking both HB-EGF and TGFα show a highly penetrant EOB phenotype that is not observed in *Rhbdf1-*null mice, suggesting that RHBDF2 compensates for the loss of RHBDF1 only during early development to facilitate EGFR signaling, including during eyelid development, through secretion of TGFα.

### RHBDF2

In vitro*,* loss of RHBDF2 has been shown to result in significantly reduced stimulated secretion of EGFR ligands, including AREG, HB-EGF, and TGFα (Siggs et al. 2014; Maretzky et al. 2013). In vivo studies in mice suggest that AREG is a *bona fide* physiological target of RHBDF2. In humans, dominant mutations in RHBDF2 cause tylosis with esophageal cancer (TOC) syndrome through a hyperactive EGFR signaling pathway. Using spontaneous (*Rhbdf2*^*cub/cub*^ and *Rhbdf2*^*cub/cub*^* Areg*^*−/−*^) (Hosur et al. 2014) and CRISPR/Cas9 (*Rhbdf2*^*TOC*^) (Hosur et al. 2017) genetically engineered mouse models, we have shown that dominant mutations in *RHBDF2* induce a hyperactive EGFR phenotype through enhanced secretion of AREG, and that genetic deletion of *Areg* in *Rhbdf2*^*cub/cub*^ mice or *Rhbdf2*^*TOC*^ mice prevents TOC. Further, shRNA-mediated silencing of *Areg* inhibits the hyperactive EGFR signaling phenotype in *Rhbdf2*^*cub/cub*^ embryonic fibroblasts (Hosur et al. 2014). Together, these studies suggest that AREG is a physiological target of RHBDF2.

## Cytosolic N-terminus and TM helix 1 of RHBDF1 are dispensable for normal growth and development

**Fig. 3:**
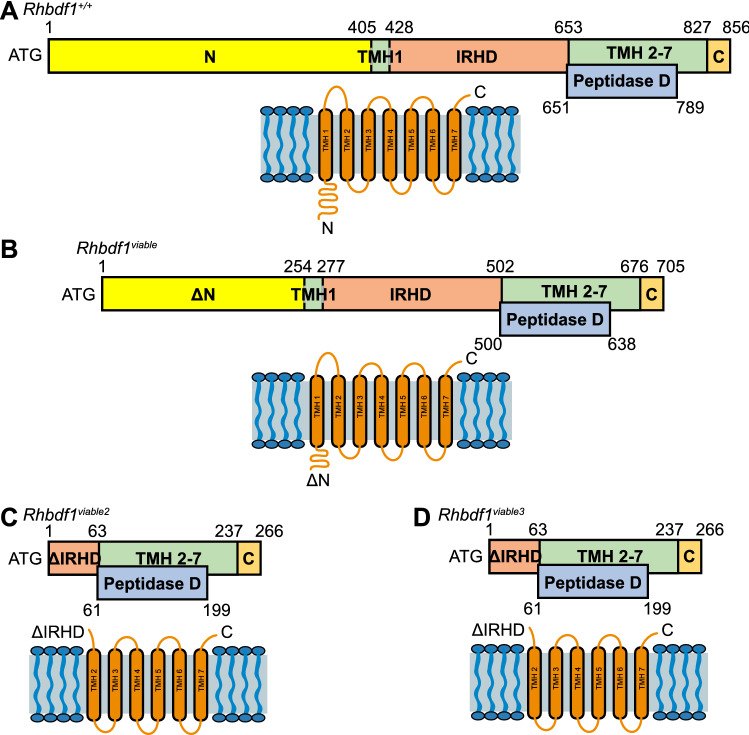
Domains of RHBDF1 gain-of-function proteins. **A** A schematic of the full-length mouse RHBDF1 protein showing the cytosolic N-terminus domain, IRHD, transmembrane helices, and the peptidase domain. **B** A schematic of the mouse RHBDF1 *viable* protein showing the loss of 151 amino acids in the N-terminal domain. **C** A schematic of the mouse RHBDF1 *viable2* protein showing the complete loss of the cytosolic N-terminal domain and the partial loss of the IRHD (Targeted KO-first allele). **D** A schematic of the mouse RHBDF1 *viable3* protein showing the complete loss of the N-terminal domain and the partial loss of the IRHD. Notably, all three mutant proteins, *viable*, *viable2*, and *viable3* retain the dormant peptidase domain (CRISPR/Cas9 generated allele)

The *Rhbdf1*^*viable*^ mutation generates an N-terminal-truncated RHBDF1 protein (ΔN1–151) (Fig. 3A, B), which rescues the severe multiorgan pathologies observed in RHBDF1-deficient mice (Fig. 2D). Notably, even in the absence of the N-terminal domain, the mutant *Rhbdf1*^*v/v*^ transcript generated by the *Rhbdf1*^*v/v*^ mutation induces enhanced secretion of EGFR ligands, suggesting that the IRHD and the TMD of RHBDF1 are sufficient to mediate EGFR signaling.

*Rhbdf1*^*viable2*^ mice, which were first generated by Li X. et al. are *Rhbdf1* homozygous mutant mice lacking exons 4–11 in the *Rhbdf1* gene (Li et al. 2015). The resulting *Rhbdf1* mutant transcripts yield two variant proteins (~ 32 and ~ 29 kDa) that each lack the entire N-terminus, the TM helix 1, and the majority of the IRHD (Fig. 3C). Like *Rhbdf1*^*viable*^ mice, *Rhbdf1*^*viable2*^ mice are healthy and fertile and do not exhibit the cardiac, brain, or growth defects observed in *Rhbdf1*-null mice. *Rhbdf1*^*viable2*^ mice are referred to by this name because of the phenotypic similarity to *Rhbdf1*^*viable*^ mice. Since the N-terminus, the TM helix 1, and the majority of the IRHD are lost in *Rhbdf1*^*viable2*^ mice, and these mice remain healthy and fertile, we reasoned that the entire N-terminus, the IRHD, and the TM helix 1 might be dispensable. Instead, we found that either of the two variant proteins, each consisting solely of TM helices 2–7, is adequate to rescue the *Rhbdf1*-null phenotype (Hosur et al. 2020). This result explains the healthy and fertile phenotypes observed in *Rhbdf1*^*viable*^ and *Rhbdf1*^*viable2*^ mice, with no brain, heart, or body weight defects.

The *Rhbdf1*^*viable2*^ homozygous mutant mice reported by Li et al. were generated using the KO-first gene disruption strategy. Nevertheless, the *Rhbdf1*^*viable2*^ mutant mice are not homozygous-null because the *Rhbdf1*^*viable2*^ mutant transcript generates truncated proteins through an alternative promoter usage and through exon skipping. Interestingly, DNA sequencing revealed that the *Rhbdf1*^*viable2*^ allele retains the *En2* splice acceptor sequence from the cassette used to generate the KO-first allele, resulting in novel mutant transcripts lacking the N-terminus, TM helix 1, and the majority of the IRHD (72%) (Hosur et al. 2020). Thus, to further validate if the N-terminus, TM helix 1, and the majority of the IRHD are dispensable, we generated *Rhbdf1*^*viable3*^ mice using CRISPR/Cas9-mediated gene editing in C57BL/6 J mice. We excised exons 4 through 13 in the *Rhbdf1* gene, resulting in mice lacking the N-terminus, the first TM helix 1, and 72% of the IRHD, but that retain TM helices 2–7 that harbor the dormant peptidase domain (Fig. 3D). We refer to these mice as viable 3 (*Rhbdf1*^*v3*^) (unpublished observations). We observed that homozygous-viable 3 (*Rhbdf1*^*v3/v3*^) mice did not display any gross deformities of major organs, which is consistent with both the *Rhbdf1*^*viable*^ (*Rhbdf1ΔN1-151*) mice and *Rhbdf1*^*viable2*^ (lacking exons 4–11) mice, and in contrast to the *Rhbdf1*^*null/null*^ mice that show multiorgan pathology and die within two to three weeks. Collectively, these studies suggest that the cytosolic N-terminus, TM helix 1, and possibly the IRHD of RHBDF1 are dispensable for normal growth and development.

## Cytosolic N-terminus and TM helix 1 of RHBDF2 regulate inflammatory signaling through TNFα secretion

**Fig. 4:**
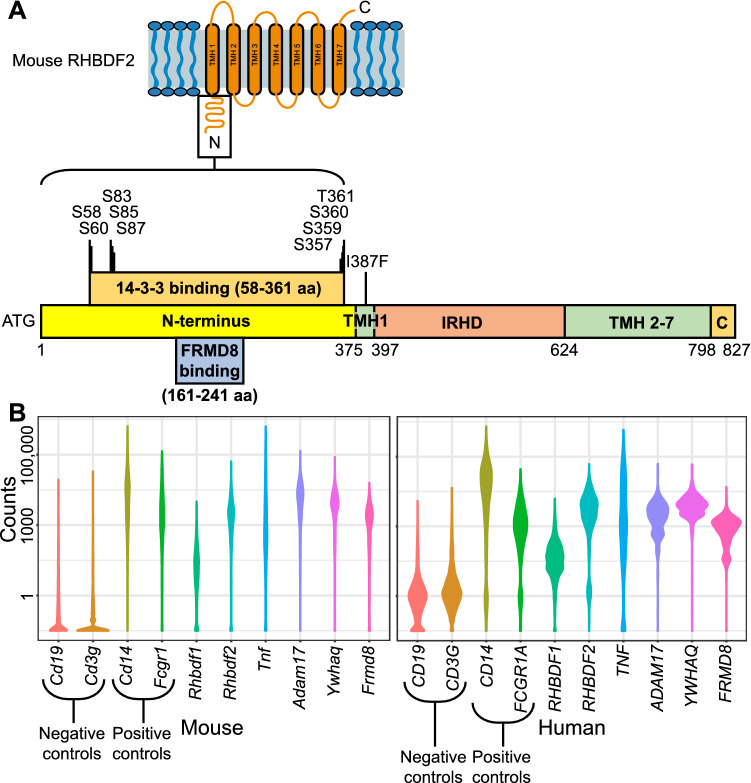
RHBDF2 binding partners and iRhoms macrophage expression. **A** A schematic of the full-length mouse RHBDF2 protein showing the 14–3-3 and FERM Domain Containing 8 (FRMD8) binding sites in the cytosolic N-terminal domain and the *sinecure* mutation I387F in transmembrane helix 1. **B** Violin plots showing expression of various mouse and human genes in macrophages. In both mouse (left panel) and human (right panel) macrophages, *Rhbdf1*, *Rhbdf2*, *Tnfa*, and *Adam17* are expressed. *Cd19* and *Cd3g* serve as negative markers, whereas *Cd14* and *Fcgr1* serve as positive controls, in macrophages

While the N-terminus and the TM helix 1 of iRhoms are dispensable for normal growth and development, they nevertheless play an important role in RHBDF2-mediated inflammatory signaling. In mice, loss of RHBDF2 significantly reduces regulated secretion of TNFα following stimulation with bacterial endotoxin lipopolysaccharide (LPS) (Hosur et al. 2014; Adrain et al. 2012; McIlwain et al. 2012; Siggs et al. 2012). Particularly, the N-terminus seems to be essential for TNFα secretion, because *Rhbdf2*^*cub*^ mice, which lack the N-terminal domain similarly to *Rhbdf2*-null mice, show significantly reduced TNFα secretion upon stimulation with LPS, demonstrating that the N-terminal domain is essential for TNFα secretion (Hosur et al. 2014). Concordantly, Cavadas et al. and Grieve et al. found that phosphorylation of RHBDF2 at the N-terminus is essential for TNFα secretion (Cavadas et al. 2017; Grieve, et al. 2017); upon stimulation with LPS, RHBDF2 serine phosphorylation and binding to 14–3–3 proteins was observed in primary macrophages. Additionally, in *Rhbdf2* KO macrophages, LPS-induced TNFα secretion was rescued by RHBDF2, but not by N-terminal-truncated RHBDF2 lacking phosphorylation sites (58–361 aa) (Fig. 4A), suggesting that RHBDF2 phosphorylation and binding to 14–3–3 proteins controls TNFα release in macrophages. In addition to binding of 14–3–3 proteins, FRMD8 has been shown to be a binding partner for RHBDF2. Künzel et al. and Oikonomidi et al. suggest that the N-terminus of RHBDF2 forms a tripartite complex with FRMD8 and ADAM17, a metalloprotease essential for ectodomain shedding of TNFα, to facilitate inflammatory signaling through stimulated secretion of TNFα (Künzel et al. 2018; Oikonomidi et al. 2018). The authors showed that stimulation of *Frmd8* KO macrophages with LPS resulted in reduced secretion of TNFα, suggesting that FRMD8-RHBDF2 interaction is necessary for TNFα secretion.

It has been suggested that the TM helix 1 of RHBDF2 is also essential for TNFα secretion in macrophages (Li et al. 2017). Like *Rhbdf2-*null mice, mice homozygous for the *sinecure* (sin) mutation, a recessive mutation in the *Rhbdf2* gene, are viable and fertile (Siggs et al. 2012). The Beutler laboratory identified *sinecure* during a forward genetic screen of mice for regulators of Toll-like receptor (TLR)-induced TNFα secretion. A single nonsynonymous mutation (A to T) in *Rhbdf2* results in conversion of isoleucine to phenylalanine at amino acid 387 (I387F) (Fig. 4A). Non-complementation studies validated that *sinecure* is a mutation at the *Rhbdf2* locus, as compound mutant *Rhbdf2*^*sin/null*^ mice and *Rhbdf2*^*sin/sin* ^*Rhbdf2-null* mice showed similar reductions in TNFα secretion following stimulation with LPS. TNFα secretion was not completely blocked, suggesting that constitutive secretion of TNFα is not affected by RHBDF2 deficiency. These data suggest that TM helix 1 of RHBDF2 is essential for stimulated secretion of TNFα.

Interestingly, the binding partners for the N-terminus of RHBDF2—14–3–3 proteins and FRMD8—seem to be dispensable for RHBDF1-mediated growth factor signaling. We recently generated *Ywhaq* (14–3–3 theta) KO mice and observed that, compared with heterozygous-null mice (*Ywhaq*^±^), homozygous-null mice (*Ywhaq*^*−/−*^) showed reduced TNFα secretion following stimulation with LPS. However, *Ywhaq*-null mice exhibit normal body weight, no postnatal lethality, nor any brain or heart defects, as observed in *Rhbdf1* KO mice. This suggests that YWHAQ could be a binding partner for RHBDF2 and that it might be essential for RHBDF2-mediated stimulated secretion of TNFα, but not for RHBDF1-mediated growth factor signaling (unpublished observations). In line with these findings, FRMD8 also seems to be essential for TNFα secretion, but not for growth factor signaling. Specifically, we generated *Frmd8* KO mice and observed that, compared with heterozygous-null mice (*Frmd8*^±^), homozygous-null mice (*Frmd8*^*−/−*^) showed significantly reduced TNFα secretion following stimulation with LPS, in accordance with the observations of Künzel et al. and Oikonomidi et al. However, *Frmd8* KO mice do not phenocopy *Rhbdf1* KO mice in terms of lower body weight, postnatal lethality, or brain and heart defects (unpublished observations). This suggests that FRMD8 could also be a binding partner for RHBDF2 and might be essential for RHBDF2-mediated stimulated secretion of TNFα, but not for RHBDF1. More importantly, RHBDF2-mediated stimulated secretion of TNFα suggests high specificity of RHBDF2 for TNFα. Even though RHBDF1 is expressed in both mouse and human macrophages (Fig. 4B), it does not compensate for the loss of RHBDF2 in regulating stimulated secretion of TNFα, suggesting that TNFα could be a specific target of RHBDF2.

## The dormant peptidase domain of iRhoms (TM helices 2–6)

*Peptidase domain of RHBDF1 is sufficient for normal growth and development.* In an in vivo screen in mice, we identified the minimal protein domain required for normal growth and development—the transmembrane peptidase domain of RHBDF1. The healthy and fertile phenotypes of *Rhbdf1*^*viable*^ (*ΔN1–151*)*, Rhbdf1*^*viable2*^, *Rhbdf1*^*viable3*^, and *Rhbdf1*^*viable*^* Rhbdf2*^*−*/−^ mice (Hosur et al. 2020) (Fig. 2D, F), with no defects in the brain, heart, or in body weight, surprisingly suggest that RHBDF2 and the N-terminus, the IRHD, and the first TM helix of RHBDF1 are dispensable for normal growth and development (Hosur et al. 2020). However, TM helices 2–6, which harbor the dormant peptidase domain of RHBDF1, are adequate and essential for survival and normal growth and development.

*Peptidase domain of RHBDF2 facilitates AREG secretion.* The *Rhbdf2*^*cub*^ spontaneous mouse mutation results from a ~ 12.5 Kb deletion in the *Rhbdf2* gene, leading to the loss of exons 2 through 6. Nevertheless, the *Rhbdf2*^*cub*^ mutant transcript generates an N-terminal-truncated protein using an in-frame ATG in exon 8 resulting in loss-of-hair and rapid wound healing phenotypes. We previously showed that the N-terminal-truncated transcript is sufficient to induce AREG secretion, leading to a hyperactive EGFR phenotype. Furthermore, using site-directed mutagenesis, we showed that mutating residues in TM helices 2, 4, and 6, which harbor the dormant peptidase domain, prevents AREG secretion, suggesting that the dormant peptidase domain of RHBDF2 is sufficient to facilitate AREG secretion, and that the N-terminal domain is dispensable for mediating the hyperactive EGFR phenotype observed in *Rhbdf2*^*cub*^ mice (Hosur et al. 2014).

## Highly conserved amino acid residues in the dormant peptidase domain of iRhoms

**Fig. 5:**
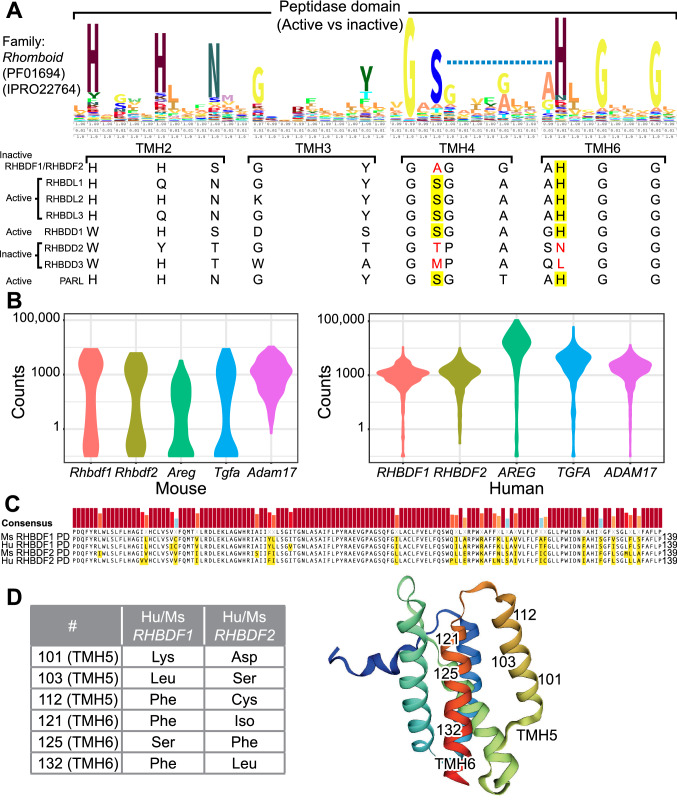
Sequence alignment of the rhomboid peptidase domain. **A** Sequence alignment of the peptidase domain of the rhomboid family of proteins showing highly conserved catalytic serine and histidine residues in transmembrane helix (TMH) 4 and TMH6, respectively. Rhomboid proteins lacking the catalytic dyad (serine and/or histidine residues) do not show protease activity (highlighted residues). **B** Violin plots showing expression of various mouse and human genes in keratinocytes. In both mouse (left panel) and human (right panel) keratinocytes, *Rhbdf1*, *Rhbdf2*, *Areg, Tgfa,* and *Adam17* are expressed. **C** Sequence alignment of mouse and human rhomboid dormant peptidase domain with non-similar residues between RHBDF1 and RHBDF2 highlighted. **D** Amino acid residues in TMH 5 and 6 that are non-similar between RHBDF1 and RHBDF2 in both human and mouse (left panel). The crystal structure of the *E. coli* rhomboid protease GlpG was used to indicate the non-similar residues in TMH 5 and 6 (right)

As noted above, the dormant peptidase domain of RHBDF2 is sufficient to induce accelerated wound healing in mice through enhanced secretion of AREG (Hosur et al. 2014). Furthermore, the survival of *Rhbdf1*^*viable2*^ and *Rhbdf1*^*viable3*^ mice indicates that the dormant peptidase domain is sufficient for survival. Nevertheless, the underlying molecular mechanisms are unclear. Here, we perform new sequence analysis of key amino acid residues in the peptidase domain of the rhomboid family using HMM Logos, a widely used tool for visualization of protein families, and uncover highly conserved amino acid residues in TM helices 2, 3, 4 and 6. We thus propose that these amino acids could be critical for RHBDF1- and RHBDF2-mediated EGFR signaling (Fig. 5A). We further propose that the dormant peptidase domain could also account for differences in substrate specificity of RHBDF1 and RHBDF2. For instance, even though both *Rhbdf1* and *Rhbdf2* are expressed in keratinocytes (Fig. 5B), RHBDF2, but not RHBDF1, selectively induces accelerated wound healing in mice through enhanced AREG secretion. Therefore, RHBDF1 and RHBDF2 have high specificity toward target proteins, which might be conferred by the peptidase domain. In addition, RHBDF2 shows high specificity for TNFα (Hosur et al. 2014; Adrain et al. 2012; McIlwain et al. 2012; Siggs et al. 2012). Despite significant sequence homology, there are several amino acids, particularly in TM helices 5 and 6, that are dissimilar in both humans and mice between RHBDF1 and RHBDF2 (Fig. 5C). Previously, it has been suggested that amino acid residues in the transmembrane domain of the substrate (e.g., TGFα, EGF) determine specificity of rhomboid proteases (Urban and Freeman 2003); however, it is likely that amino acid residues in TM helices 5 and 6 of the peptidase domain could also account for differences in specificity. Because TM helix 5, which tilts its top ~ 35° laterally from the enzyme core (Fig. 5D), acts as the substrate gate (Baker et al. 2007), differences in amino acid residues in TM helix 5 could govern substrate specificity. However, future studies are needed to more thoroughly define the role of the dormant peptidase domain in conferring specificity for targets.

## How do iRhoms regulate secretion of EGFR ligands and TNFα?

The underlying mechanisms are still emerging, but two hypotheses have been proposed based on available data:

### ADAM17 hypothesis

iRhoms have been shown to regulate maturation, trafficking, and activation of ADAM17, a metalloprotease required for ectodomain shedding of EGFR ligands and TNFα (Adrain et al. 2012; McIlwain et al. 2012; Maretzky et al. 2013). Thus, according to this hypothesis, mice lacking *Rhbdf1* or *Rhbdf2* or both together fail to promote ADAM17 activity, thereby leading to significantly reduced secretion of EGFR ligands and TNFα following stimulation with phorbol ester or LPS. However, genetic evidence deduced from the mouse models that we and others have generated argue against this hypothesis. First, the necessity of RHBDF2 for ADAM17 maturation and trafficking raises an obvious question as to why *Rhbdf2* KO mice do not phenocopy *Adam17* KO mice. Notably, whereas *Adam17* KO results in embryonic or perinatal lethality (Veit 2019), *Rhbdf2* KO mice are viable and fertile. To this end, Issuree et al. (Issuree et al. 2013) suggested that RHBDF1 compensates for the loss of RHBDF2 in *Rhbdf2* KO mice and facilitates ADAM17 maturation and trafficking, and hence *Rhbdf2* KO mice do not phenocopy *Adam17* KO mice. Consistent with the Issuree et al. study, Li et al. (2015) showed that, whereas *Rhbdf1* and *Rhbdf2* single KO mice are viable and fertile, *Rhbdf1/Rhbdf2* double KO mice phenocopy *Adam17* KO mice, exhibiting perinatal lethality, open eyelids at birth, and heart valve defects. Li et al.’s findings are in direct conflict with the results of a previous study by Christova et al. (2013), who found that *Rhbdf1* single KO mice have multiorgan pathologies, and *Rhbdf1*:*Rhbdf2* double KO mice show early embryonic lethality. To try to resolve this discrepancy, we found that both the *Rhbdf1* single KO mice and the *Rhbdf1/Rhbdf2* double KO mice generated by Li et al. are indeed not null for RHBDF1 as they retain residual RHBDF1 functional activity (Hosur et al. 2020). This brings into question the notion as to whether RHBDF1 compensates for the loss of RHBDF2 in regulating ADAM17 maturation and trafficking, and the association in general between iRhoms and ADAM17 maturation, trafficking, and activation. Second, transcriptome data suggest that macrophages (human and mouse) express both *Rhbdf1* and *Rhbdf2* (Fig. 4B). According to the ADAM17 hypothesis, loss of RHBDF2 is compensated by RHBDF1, and vice versa. However, *Rhbdf2* KO macrophages show significantly reduced stimulated secretion of TNFα (Hosur et al. 2014; Adrain et al. 2012; McIlwain et al. 2012; Siggs et al. 2012), even though *Rhbdf1* is expressed in *Rhbdf2*-null macrophages, arguing against the iRhoms-ADAM17 hypothesis. Third, *Rhbdf2-null* mice show reduced stimulated secretion of EGFR ligands, including AREG and TGFα, in keratinocytes. It has been suggested that loss of RHBDF2 fails to promote ADAM17 maturation, trafficking, and activity, leading to the reduction in stimulated secretion of EGFR ligands. Again, according to the ADAM17 hypothesis, RHBDF1, which is abundantly expressed in keratinocytes (Fig. 5B), compensates for the loss of RHBDF2. However, contrary to this prediction, *Rhbdf2*-null keratinocytes show significantly reduced stimulated secretion of EGFR ligands. Lastly, and more importantly, this hypothesis does not account for target specificity of RHBDF1 and RHBDF2. For instance, GOF mutation in *Rhbdf2* (*Rhbdf2*^*cub*^), but not in *Rhbdf1* (*Rhbdf1*^*viable*^), selectively regulates AREG secretion to induce loss-of-hair and wound healing phenotypes. Therefore, it seems unlikely that iRhoms regulate secretion of EGFR ligands and TNFα through direct regulation of ADAM17 maturation, trafficking, and activation.

### Target trafficking hypothesis

According to this hypothesis, RHBDF1 regulates secretion of EGFR ligand TGFα through delivery of pro-TGFα to the plasma membrane, where pro-TGFα undergoes ectodomain shedding by ADAM17 to release TGFα. In breast cancer cell lines, Li J. et al. showed that RHBDF1 is an essential component of the protein trafficking machinery involving clathrin-coated vesicles (Li et al. 2018). Following stimulation with the G-protein-coupled receptor agonist Sphingosine 1 Phosphate (S1P), RHBDF1 participates in clathrin uncoating of vesicles to deliver pro-TGFα to the cell surface. Specifically, RHBDF1 interacts with a clathrin-coated vesicle protein auxilin-2 to recruit Heat shock cognate protein (HSC70) to the vesicles to initiate clathrin uncoating. Furthermore, siRNA-mediated silencing of *RHBDF1* inhibits the interaction between HSC70 and auxilin-2, significantly reducing TGFα secretion by preventing uncoating of clathrin and delivery of pro-TGFα to the plasma membrane for ectodomain shedding. Although in vivo validation is required to further support the target trafficking hypothesis, these findings could help address certain unanswered questions, such as the observation that *Rhbdf2* KO macrophages demonstrate reduced stimulated secretion of TNFα. According to the target trafficking hypothesis, RHBDF2 deficiency does not affect ADAM17 activity, but due to target specificity (RHBDF2 for TNFα), RHBDF2 deficiency in macrophages, regardless of *RHBDF1* expression, suppresses TNFα secretion.

## Concluding remarks

iRhoms are characterized by a cytosolic N-terminal domain, a luminal IRHD, and a transmembrane dormant peptidase domain. Since the initial discovery in *Rhbdf2*^*cub*^ mice that in the absence of the cytosolic N-terminal domain, the dormant transmembrane peptidase domain of RHBDF2 is sufficient to regulate EGFR signaling through secretion of EGFR ligand AREG, a substantial amount of literature has revealed the biological functions of iRhoms domains. Furthermore, the normal development and survival of *Rhbdf1*^*viable3*^ mice demonstrates that whereas the N-terminal domain and the IRHD are dispensable for viability and fecundity, the loss of the transmembrane dormant peptidase domain is associated with developmental defects, indicating that the peptidase domain is essential and is sufficient to regulate the secretion of diverse EGFR ligands. Although in vitro biochemical assays indicate some redundant functions for RHBDF1 and RHBDF2 in controlling secretion of various EGFR ligands, mouse genetic studies reveal unique signaling pathways and distinct client proteins for iRhoms. Additionally, uncovering the pathological role of iRhoms in epithelial cancers, inflammation, and skin diseases suggests iRhoms as potential therapeutic targets. Particularly, since ADAM17 inhibition to block secretion of EGFR ligands, including AREG, is associated with severe adverse effects (Ieguchi and Maru 2016), identification of selective inhibitors of the dormant peptidase domain—although challenging—could lay the foundation for the development of more selective and effective therapeutics targeting iRhoms to abrogate multiple pathogenic signaling pathways.

## Highlights

- Rhomboid proteases, first discovered in *Drosophila*, are intramembrane serine proteases. Members of the rhomboid protein family lacking protease activity are known as inactive rhomboids (iRhoms) or pseudoproteases.
- Both spontaneous and genetically engineered mouse models of iRhoms have been critical tools to explore the molecular and cellular functions of key iRhom protein domains—the cytosolic N-terminus, and the transmembrane dormant peptidase domain—in development and disease.
- The N-terminus and the dormant peptidase domain have opposing roles. While the N-terminus negatively regulates EGFR signaling, the dormant peptidase domain stimulates EGFR signaling when not suppressed by the N-terminus.
- RHBDF1 and RHBDF2 have both discrete and overlapping functions during development. For survival and normal growth, the dormant peptidase domain of RHBDF1 is adequate to compensate for the loss of the N-termini of iRhoms and for the complete loss of RHBDF2.
- The N-terminal domain and TM helix 1 of RHBDF2 are essential for TNFα secretion.
- The iRhom homology domain (IRHD) of RHBDF1 appears to be dispensable for survival and/or ligand secretion, but the role of the IRHD of RHBDF2 is unknown*.*

## Outstanding questions

- *Rhbdf1* KO mice die of brain hemorrhage and cardiac fibrosis. What are the physiological substrates of RHBDF1 and the underlying signaling pathways that maintain brain and heart function? Addressing these questions may have implications for treating neurological disorders, such as Alzheimer’s and Parkinson’s, as well as cardiac abnormalities.
- TM helices 2–6, which harbor the dormant peptidase domain, of iRhoms are essential for stimulated secretion of EGFR ligands. Which amino acid residues in the dormant peptidase domain regulate secretion in vivo? These findings will help in rational drug design of potential novel treatments for cancer and skin diseases.
- iRhoms have overlapping functions only during developmental stages. Why does RHBDF2 not rescue multiorgan pathology, including brain hemorrhage and cardiac fibrosis, in *Rhbdf1-null* mice? Is RHBDF2 not expressed in the brain and heart during later stages, i.e., postnatal days?
- There is a need to understand tissue-specific regulation of EGFR ligand secretion by iRhoms. Mutations in the N-terminus (*Rhbdf2*^*TOC*^) or loss of the entire N-terminus (*Rhbdf2*^*cub*^) in RHBDF2 enhance AREG secretion to cause a hair loss phenotype. However, mutations in the N-terminus (*Rhbdf1*^*viable*^) or loss of the entire N-terminus (*Rhbdf1*^*viable3*^) in RHBDF1 do not result in a similar phenotype.

*Note* Tissue-specific gene expression data were obtained from the ARCHS^4^ database, which provides access to gene counts from HiSeq 2000, HiSeq 2500, and NextSeq 500 platforms for human and mouse experiments from GEO and SRA (Lachmann et al. 2018). We downloaded expression files (gene-level) for mouse (mouse_matrix_v10.h5) and human ((human_matrix_v10.h5) and selected the samples with tissue annotation from metadata as macrophages (Fig. 4A) and keratinocytes (Fig. 5B).
